# Pharmacokinetic Monitoring of JAK Inhibitor and Tacrolimus for Safe and Effective Management of Graft‐Versus‐Host Disease After Pediatric Liver Transplantation

**DOI:** 10.1111/petr.70187

**Published:** 2025-10-02

**Authors:** Keisuke Sawada, Pooja Khandelwal, Stella M. Davies, Anna Crooker, Tomoyuki Mizuno, Akihiro Asai

**Affiliations:** ^1^ Department of Pediatrics University of Cincinnati College of Medicine Cincinnati Ohio USA; ^2^ Division of Immunobiology Cincinnati Children's Hospital Medical Center Cincinnati Ohio USA; ^3^ Medical Scientist Training Program University of Cincinnati College of Medicine Cincinnati Ohio USA; ^4^ Division of Bone Marrow Transplantation and Immune Deficiency Cincinnati Children's Hospital Medical Center Cincinnati Ohio USA; ^5^ Division of Gastroenterology, Hepatology, and Nutrition Cincinnati Children's Hospital Medical Center Cincinnati Ohio USA; ^6^ Division of Translational and Clinical Pharmacology Cincinnati Children's Hospital Medical Center Cincinnati Ohio USA

**Keywords:** GVHD, pediatric liver transplantation, pharmacokinetics, therapeutic drug monitoring

## Abstract

**Background and Aim:**

Though liver transplantation (LT) is effective for pediatric patients with end‐stage liver disease, it is associated with complications such as graft‐versus‐host disease (GVHD). In part due to its rarity and lack of standardized therapy, post‐LT GVHD is associated with a high mortality rate. While ruxolitinib (a JAK inhibitor) is approved for use in steroid‐refractory GVHD, its safety/efficacy post‐LT is undefined. Thus, this case study reports the use of ruxolitinib in a pediatric patient with post‐LT GVHD.

**Methods:**

A 5‐month‐old male underwent a deceased donor left lateral segment LT with an induction regimen of corticosteroid and tacrolimus. Four weeks post‐LT, the patient developed skin GVHD. Ruxolitinib was administered in combination with tacrolimus and posaconazole, both capable of drug–drug interactions with ruxolitinib via modulation of CYP3A4 activity. A pharmacokinetic (PK) study was conducted to evaluate the use of ruxolitinib to induce GVHD remission.

**Results:**

The ruxolitinib PK study revealed sufficient oral clearance and effective maximum serum concentration. Dosages of ruxolitinib, tacrolimus, and posaconazole were adjusted throughout the course of treatment based on GVHD severity (ruxolitinib) or their whole blood/plasma concentrations (tacrolimus and posaconazole). By 18 weeks after ruxolitinib initiation, skin GVHD lesions resolved and clinical remission was induced. The liver graft function was normal when assessed 40 months after the initial presentation of GVHD.

**Conclusions:**

This case illustrates the safe and effective PK‐validated use of ruxolitinib with tacrolimus and posaconazole to successfully induce remission of post‐LT GVHD in a pediatric patient.

**Abbreviations**
allo‐HSCT: allogeneic hematopoietic stem cell transplantation
GVHD: graft‐versus‐host disease
HLA: human leukocyte antigen
LC–MS/MS: liquid chromatography–tandem mass spectrometry
LT: liver transplantation
PK: pharmacokinetic

## 1 Introduction

An estimated 500–600 cases of pediatric liver transplantation (LT) are performed each year in the United States [1]. However, recipient mortality remains a significant issue, as death occurs in 9.8% by 5 years post‐LT [1]. Thus, there is an ongoing need for improvements in postoperative management for better patient survival.

Among various post‐LT complications, graft‐versus‐host disease (GVHD) has an extremely high mortality rate, with estimations to be as high as 85% [2, 3]. Post‐LT GVHD occurs when the donor's immunocompetent cells, migrating with the donor's liver, recognize the recipient antigens as non‐self, and trigger a persistent immune response within recipient tissues [4]. GVHD typically presents 2–6 weeks after LT and commonly manifests as fever, rash, and diarrhea [3, 5]. Because of its rarity, with an incidence of 0.5%–1% in adults and less than 30 reported cases in pediatric patients, no standard treatment regimen has been established [2, 3, 5, 6, 7]. Enhancing immunosuppressive therapy using steroids and tacrolimus (a calcineurin inhibitor) is often used as first‐line treatment; however, there are reports indicating caution against extreme immunosuppression [1, 3, 5, 6, 7]. This is due in part to serious opportunistic infections being the most common cause of death in post‐LT GVHD, with invasive fungal (aspergillosis and candidiasis) infection as the dominant cause [2, 3, 5]. Hence, there is an unmet need for establishing effective and safe therapy for post‐LT GVHD.

Limited reports have suggested promising results using ruxolitinib, in combination with a modified dose of tacrolimus, in both adult and pediatric subjects with post‐LT GVHD [5, 8, 9]. Ruxolitinib is a selective inhibitor of Janus kinase (JAK) 1 and 2, enzymes that activate Signal Transducers and Activators of Transcription (STAT) proteins [10]. JAK–STAT signaling in lymphocytes is critical in the activation of host immune responses and plays a significant role in the pathogenesis of GVHD [11]. Ruxolitinib is FDA‐approved for the treatment of steroid‐refractory acute GVHD in patients 12 years and older [12]. However, adverse events are reported, including bone marrow suppression and hepatotoxicity in pediatric patients treated with ruxolitinib, and thus we aimed to establish methods to mitigate toxicity in inducing remission in post‐LT GVHD for pediatric patients [13].

Here, we report a case of successful GVHD remission in a pediatric recipient of LT using alemtuzumab (Campath), ruxolitinib (Jakafi), and tacrolimus (Prograf), while preventing serious fungal infection with posaconazole. Both ruxolitinib and tacrolimus are metabolized via CYP3A4, and posaconazole is a strong CYP3A4 inhibitor; however, the ruxolitinib PK study indicated the safety and efficacy of the therapeutic regimen, ultimately leading to successful remission of GVHD while mitigating serious fungal infection and sepsis [14, 15, 16].

## 2 Methods

### 2.1 Graft‐Donor Matching

Human leukocyte antigen (HLA) typing was performed using quantitative reverse transcription polymerase chain reaction, reverse sequence‐specific oligonucleotide probes, and next generation sequencing. Mismatch calls were based on shared or dissimilar antigen recognition domains. Crossmatching analysis was performed via flow cytometry. HLA antibody screening was performed using Luminex single‐antigen bead methodology. Both crossmatching and antibody screenings were performed 1 day post‐LT. Chimerism was monitored by measuring short tandem repeat markers on circulating T cells.

### 2.2 Therapeutic Drug Monitoring

Tacrolimus and posaconazole concentrations were monitored as part of the routine clinical care. Whole blood tacrolimus concentrations were measured via a chemiluminescent microparticle immunoassay, while posaconazole plasma concentrations were measured using liquid chromatography–tandem mass spectrometry.

### 2.3 Ruxolitinib PK Analysis

For the ruxolitinib PK assessment, a sparse sampling strategy was utilized using three time points (pre‐dose and 180 and 360 min after dose) at a steady state. Ruxolitinib plasma concentrations were measured using the validated LC–MS/MS method [17]. Individual PK parameters were estimated using Bayesian estimation with a published ruxolitinib PK model [18]. The allometric body weight scaling was used to account for body size with a power coefficient of 0.75 for clearances and 1 for volumes of distribution, as described previously [19]. The 2‐compartment model parameter estimates (mean ± SD) used as the Bayesian prior were 21.4 ± 8.38 L/h/70 kg for clearance (CL/F), 56.3 ± 9.18 L/70 kg for the central volume of distribution (Vc/F), 2.5 L/h/70 kg for intercompartmental clearance (Q/F), 10.8 ± 10.97 L/70 kg for the peripheral volume of distribution (Vp/F), 4.12 ± 3.09 h^−1^ for absorption rate constant (Ka), and 0.0545 h for lag time. No inter‐individual variability was set for Q/F and lag time. Additionally, Vp/F was fixed to the population mean value due to sparse sampling. The Bayesian analysis was performed using MwPharm++ (ver. 2.4, Mediware, Czech Republic). The PK parameter estimate results were scaled to 15 kg patients using the allometric scaling to compare them with our previous report [17].

## 3 Case

We present a case of a 3‐year‐old male who was diagnosed with biliary atresia at 7 weeks of age after developing jaundice and hepatomegaly. At 8 weeks of age, Kasai portoenterostomy was performed; however, his jaundice worsened and he developed intractable ascites. A deceased donor left lateral segment LT was performed at 5 months of age with an induction regimen of corticosteroid and tacrolimus. The posttransplantation course was complicated by transient intestinal pneumatosis but was otherwise uneventful until 4 weeks post‐LT when he developed a maculopapular erythematous rash, darkened and sandpaper‐like in appearance, on his arms, back, and trunk. Given the increased donor T‐cell chimerism (14.41%) and skin histopathology exhibiting vacuolar interface dermatitis with necrotic keratinocytes also present in hair follicles, a diagnosis of acute skin GVHD grade 2 was made. Notably, there were no signs of involvement at other sites. A 5‐day course of alemtuzumab was given as an initial lymphodepleting agent, followed by intensification therapy with ruxolitinib. Tacrolimus was continued, and methylprednisolone was gradually weaned. Additionally, fluconazole was switched to posaconazole for enhanced anti‐fungal prophylaxis. Given the risk of drug toxicity caused by posaconazole interacting with ruxolitinib and tacrolimus metabolism, a pharmacokinetic (PK) study was performed. Here, a sparse sampling strategy was chosen given its previous application in both pediatric and adult populations [17, 20]. Notably, sparse sampling has been reviewed as well‐established in the infant population for other drugs, and when combined with Bayesian estimation, allows for the conservation of blood volume (critical for infants like our case) by using a population PK model as a priori information [21].

## 4 Results

HLA typing revealed two mismatches on HLA‐A, ‐B, ‐C, ‐DRB1, and ‐DPB1. One day post‐LT, additional allograft‐recipient compatibility was assessed; crossmatching results were negative, while HLA antibody analysis revealed no donor‐specific antibodies. Donor T‐cell chimerism was 14.41% when GVHD rashes first appeared and peaked at 83.85% 10 weeks post‐LT.

**FIGURE 1:**
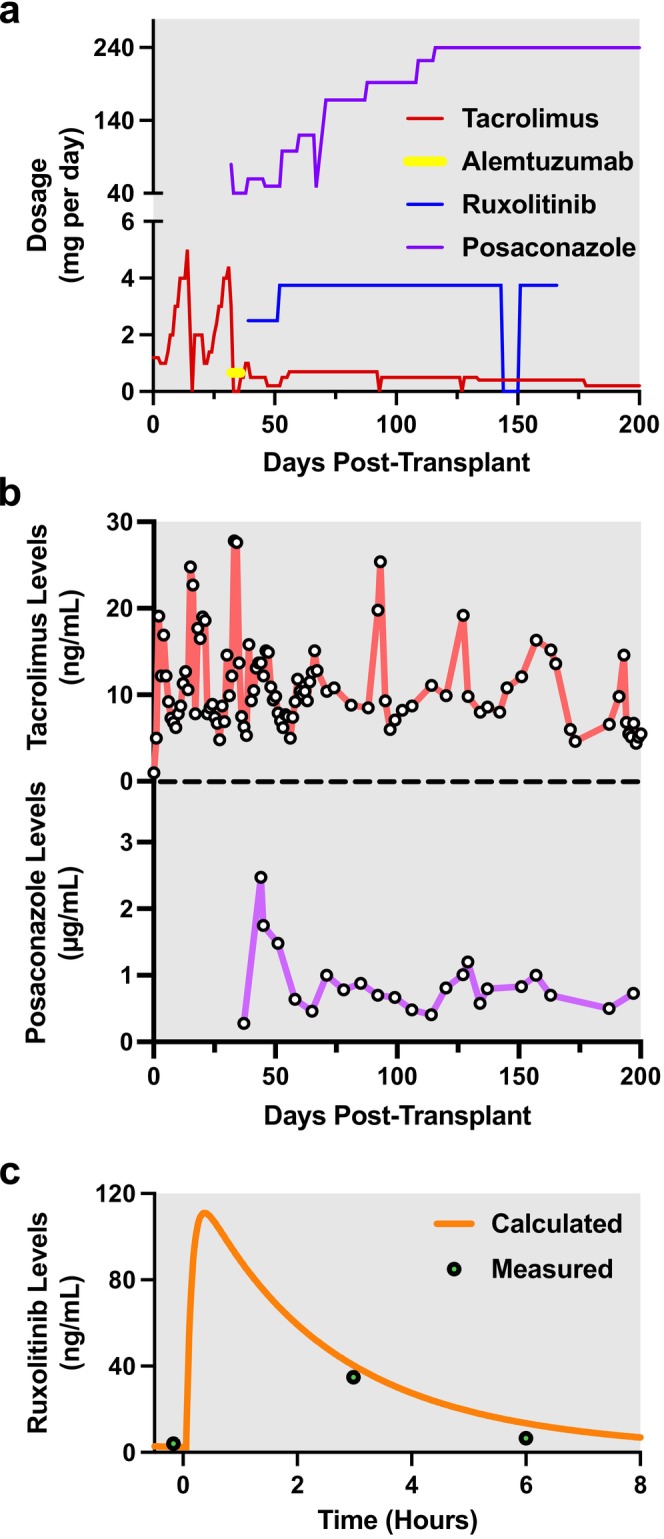
Pharmacokinetic study of ruxolitinib to assess efficacy/safety of treatment regimen in relation to dosage. (a) Ruxolitinib, tacrolimus, posaconazole, and alemtuzumab dosage. Dosages are reported as total mg of medication taken per day. (b) Tacrolimus (whole blood) and posaconazole (plasma) concentrations during the first 200 days post‐LT. (c) Ruxolitinib PK profile estimated with Bayesian estimation using measured concentrations.

Tacrolimus was started 12 h post‐LT with the initial dosage of 0.6 mg (0.1 mg/kg) twice daily (Figure 1A; red line). Upon confirmation of skin GVHD, posaconazole (loading dosage of 6 mg/kg intravenous twice daily for 2 days followed by 6 mg/kg intravenously once daily; switched to enteric administration 53 days post‐LT) and alemtuzumab (0.1 mg/kg subcutaneous once daily) were initiated (Figure 1A; purple and yellow lines, respectively). We previously found that short‐term (2–6 days) alemtuzumab course results in a high response rate for GVHD after allogeneic hematopoietic stem cell transplantation (allo‐HSCT) in pediatric patients [22]. However, alemtuzumab monotherapy was associated with 33% treatment failure among those who initially showed a good response [22]. Thus, we decided to initially treat with alemtuzumab as a lymphodepleting agent to induce remission as soon as possible, followed by intensifying treatment with ruxolitinib to overcome the severe acute GVHD. Three days after completion of the 5‐day alemtuzumab course, ruxolitinib was started with an initial dosage of 1.25 mg twice daily (Figure 1A; blue line). Tacrolimus (whole blood) and posaconazole (serum) concentrations were continuously monitored (Figure 1B). Tacrolimus and posaconazole dosages were adjusted throughout the course of treatment with a target trough concentration of 8–14 ng/mL and > 0.7 μg/mL, respectively. Specifically, tacrolimus dose adjustments were made reactively (e.g., after elevation of levels on days 15–16, 18–21, 33–34, 93, or 127 post‐LT, administration was either paused or decreased), while those of posaconazole were made proactively to maintain target trough concentration (Figure 1A,B).

Thirteen days after starting ruxolitinib, the dose was increased to 1.25 mg three times daily due to worsening GVHD‐associated rash. We have previously found that the patient's age and route of administration significantly impact ruxolitinib oral clearance [17]. Specifically, if the patient is < 2 years of age or if ruxolitinib is administered as a crushed tablet through a feeding tube, there is increased ruxolitinib clearance or lower bioavailability in patients with GVHD [17]. Our patient met both of these criteria. In addition to these factors, given coadministration with tacrolimus and posaconazole, which may also impact metabolism, a PK study of ruxolitinib was performed 21 days after increasing the ruxolitinib dosage (Figure 1C). For our patient, aged 6 months and weighing 6.4 kg at the time of the PK study, the ruxolitinib oral clearance was 3.91 L/h (allometrically scaled was 7.4 L/h/15 kg), central volume of distribution (Vc/F) was 9.98 L (23.4 L/15 kg), intercompartmental clearance (Q/F) was 0.42 L/h (0.79 L/h/15 kg), peripheral volume of distribution (Vp/F) was 0.99 L (2.3 L/15 kg), absorption rate constant (Ka) was 10.15 h^−1^, and elimination half‐life was 2.35 h. Together, these results ensured that the increased dosage (1.25 mg three times daily) was indeed safe and necessary to induce GVHD remission.

Pancytopenia, severe thrombocytopenia, and moderate anemia developed while on alemtuzumab and ruxolitinib, but were managed clinically (multiple platelets and red blood cell transfusions) without major consequences. Epstein–Barr virus was detected at a low copy level 7 weeks post‐LT without clinical manifestation of posttransplant lymphoproliferative disorder. Norovirus was first detected after initiation of the alemtuzumab course prior to ruxolitinib administration, and he subsequently suffered from persistent norovirus enterocolitis and experienced malabsorption and poor growth, though it eventually resolved. He also developed reactive airway disease 70 days after cessation of ruxolitinib (clinical symptoms and lung CT images were not compatible with lung GVHD), requiring home oxygen therapy for several months with daily corticosteroid inhalation and azithromycin prophylaxis. Severe tacrolimus toxicity (e.g., seizure) did not occur, and serum creatinine levels were stable throughout the course of treatment. Importantly, serious opportunistic fungal infection was prevented. Given that GVHD significantly increases the risk of infections, which itself is a significant risk factor for death in pediatric LT, effective posaconazole use to prevent fungal infection was vital for the survival of this patient [2, 3, 5]. Together, our PK‐validated therapeutic drug monitoring allowed for the mitigation of acute life‐threatening adverse events of combinatory therapy of ruxolitinib with tacrolimus and posaconazole.

Two days after completion of the 5‐day alemtuzumab course, the GVHD rash stopped spreading. Ruxolitinib induced gradual resolution of the rash by 23 weeks post‐LT and was discontinued based on the resolution of skin GVHD (Figure 1A, Table 1). Critically, no skin activity of GVHD has been observed once in remission (Table 1), and liver graft function stayed normal without episodes of rejection for 44 months after the presentation of GVHD.

**TABLE 1:** Signs/symptoms of skin GVHD upon liver transplantation.

Days post‐LT	Signs of skin GVHD	Posaconazole dosage	Tacrolimus dosage	Ruxolitinib dosage
27	Maculopapular erythematous rash to arms, back and trunk (confluent on extremities). Parts darkened and sandpaper in appearance.	0 mg	1.5 mg BID	0 mg
32	GVHD confirmed via skin biopsy.	40 mg BID	1.5 mg BID	0 mg
38	New papules on palms and soles.	40 mg QD	0.5 mg BID	0 mg
43	Rash is resolved.	60 mg QD	0.5 mg QD	1.25 mg BID
45	Mild increased macular rash on back of legs, cheeks and nasal bridge.	60 mg QD	0.5 mg QD	1.25 mg BID
46	New rash between eyebrows/on cheeks and at back of upper thighs.	50 mg QD	0.5 mg QD	1.25 mg BID
50	Eyelids with mild erythema. Very mildly erythematous papules over both ankles and dorsum of feet	50 mg QD	0.2 mg QD	1.25 mg BID
68	Rash over chest/back/arms/legs is stable.	0 mg	0.7 mg QD	1.25 mg TID
100	Worsening macular erythematous rash at hands, legs, thighs, and feet. Leathery skin on thighs. One small papule on the gum.	64 mg TID	0.5 mg QD	1.25 mg TID
107	Rash improved. No lesion in mouth.	64 mg TID	0.5 mg QD	1.25 mg TID
165	Rash is almost resolved.	80 mg TID	0.2 mg BID	1.25 mg TID
228	No visible activity of skin GVHD.	80 mg TID	0.2 mg QD	0 mg

Abbreviations: BID, twice a day; ND, no data (data not collected); QD, once a day; TID, three times a day.

## 5 Discussion

Our study represents the safety and efficacy of ruxolitinib when used in combination with tacrolimus and posaconazole for a pediatric case of post‐LT GVHD. Though combinatory therapy of ruxolitinib and tacrolimus for post‐organ transplant GVHD is reported, all cases describe their use in adults [8, 9, 23]. Additionally, the metabolism of both tacrolimus and ruxolitinib in pediatric patients differs from that of adults, and both are impacted by co‐medication with a CYP3A4 inhibitor [24, 25]. Thus, the impact of age and co‐medication with posaconazole in a pediatric patient having undergone transplant of the organ responsible for their metabolism (i.e., liver) is unknown [26]. Here, our patient showed ruxolitinib CL/F comparable with those of pediatric patients with post‐allo‐HSCT GVHD co‐administered with an azole, suggesting that LT may not significantly modify the effects of azole co‐administration on ruxolitinib metabolism [17]. Together, given the combination of ruxolitinib, tacrolimus, and azole is commonly co‐administered in adults with GVHD, further studies are warranted to optimize the use of such combinatory therapy for post‐LT GVHD [9, 12].

It is important to note that the alemtuzumab course was effective in stopping the initial spread of skin GVHD; however, alemtuzumab can be insufficient for the induction of remission. Therefore, we chose to add ruxolitinib to the standard tacrolimus because it is effective in inducing remission and has a lower incidence of adverse infectious complications, compared to repeating alemtuzumab treatment, which can cause fatal opportunistic infections [13, 22].

We previously reported ruxolitinib PK results in pediatric post‐allo‐HSCT GVHD, in which we utilized a 1‐compartment model [17]. Here, we found that the 1‐compartment model did not adequately capture the observed concentrations for this individual, possibly due to the sparse sampling design used for this patient and insufficient flexibility of the model [27]. Therefore, we utilized a 2‐compartment model that was developed and externally validated using extensive PK data from multiple clinical trials [18]. This model provided a better fit to the observed concentrations and a more reasonable *C*_max_ prediction (115 ng/mL by 2‐compartment model vs. 32 ng/mL by 1‐compartment model), which aligns with prior findings given the patient's dose of 0.2 mg/kg TID. Specifically, in adult populations observed *C*_max_ values range from 110 to 340 ng/mL for a 10–25 mg single dose, corresponding to approximately 0.14–0.36 mg/kg, which our calculated *C*_max_ value falls within [25]. Given that the typical *T*_max_ for ruxolitinib is 0.5–2 h post‐dose, the PK estimates using the 2‐compartment model with allometric scaling may have been more optimal for this particular patient due to the limited sampling points (0, 3, and 6 h) used for smaller patients (< 10 kg body weight), compared to the time points (0.5, 1, 2, 4, and 6 h) used in our prior study for larger children (> 10 kg) [17].

Finally, in the limited cases to date that report ruxolitinib use in post‐solid organ transplant GVHD, its use was associated with a decrease in donor T‐cell chimerism [8, 9, 23]. However, in our case, donor T‐cell chimerism was elevated (> 80%) throughout the course of treatment and remained high (36.53%–81.06%) even after GVHD rash had resolved. Interestingly, though chimerism was monitored at least once a week until skin GVHD had started improving, its value did not correlate with GVHD‐specific symptoms (i.e., skin lesion activity). Therefore, we propose that donor T‐cell chimerism may only be helpful for the diagnostic process, not for GVHD disease monitoring. Donor T‐cell chimerism is not uncommon after LT, and high levels of donor T‐cell chimerism, especially in CD8^+^ T cells, have been associated with acute GVHD [28]. Given the importance of JAK/STAT signaling in T‐cell effector functions, ruxolitinib may have induced remission of GVHD via reduction of their effector function (e.g., cytokine production) in our case. Further mechanistic interrogation of how ruxolitinib is beneficial in post‐LT GVHD, and the clinical parameters that may dictate its success in inducing remission, is warranted.

## 6 Conclusion

Previous reports introduce several approaches (e.g., increased immunosuppression, anti‐lymphocyte therapy, anti‐interleukin‐2 receptor antibodies) for post‐LT GVHD care, yet most show limited effectiveness [5]. Here, we used a personalized therapeutic regimen based on previous reports in adult acute GVHD [29]. However, toxicity from drug–drug interaction remains a strong concern because optimal dosages are not well established in pediatric patients. Conducting a PK study with model‐informed Bayesian analysis, we successfully demonstrated that the patient's ruxolitinib PK was consistent with previous reports and, moreover, the safety of its dosage when administered with tacrolimus and posaconazole. Together, our case highlights the use of a limited sampling strategy PK assessment to validate a novel therapeutic regimen to treat post‐LT GVHD safely and effectively.

## Conflicts of Interest

The authors declare no conflicts of interest.

## Data Availability

The data that support the findings of this study are available on request from the corresponding author. The data are not publicly available due to privacy or ethical restrictions.
